# Determinants of workplace safety towards SARS-Cov-2 and combating COVID-19 among non-healthcare workers in Hong Kong, Nanjing and Wuhan, China

**DOI:** 10.1038/s41598-022-19195-4

**Published:** 2022-09-09

**Authors:** Lap Ah Tse, Priscilla Ming Yi Lee, Dongming Wang, Yan Li, Shuyuan Yang, Shoulin Wang, Janice Ying Chui Lau, Tangchun Wu, Hongbing Shen, Xiaoming Ji, Weihong Chen

**Affiliations:** 1 JC School of Public Health and Primary Care, The Chinese University of Hong Kong, School of Public Health Building, Prince of Wales Hospital, Sha Tin, Hong Kong Special Administrative Region China; 2 School of Public Health, Tongji Medical College, Huazhong University of Science and Technology, Wuhan, 430030 Hubei China; 3 School of Public Health, Nanjing Medical University, 101 Longmian Avenue, Jiangning District, Nanjing, 211166 China; 4 Stanley Ho Centre for Emerging Infectious Diseases, The Chinese University of Hong Kong, Prince of Wales Hospital, Sha Tin, Hong Kong Special Administrative Region China

**Keywords:** Public health, Epidemiology

## Abstract

There has been no validated tool to assess workplace infection control towards SARS-Cov-2 in non-healthcare industries. In this first year survey during 07/2020–04/2021, 6684 workers were recruited from varied non-healthcare settings of Hong Kong, Nanjing and Wuhan of China and responded standard questionnaires containing information of prevention measures and policies implemented by companies and personal preventive behaviour towards infection control. All participants were randomly stratified into two sub-samples as training and validation sample. Workplace safety index towards SARS-Cov-2 (WSI-SC2) was developed and validated using exploratory factor analysis (EFA) and confirmatory factor analysis (CFA). We identified 14 manifest variables in WSI-SC2, with three sub-indices named “Workplace infection control measures and prevention”, “Company occupational safety and health management and commitment” and “Worker’s personal preventive behavior and awareness towards infectious control”. WSI-SC2 obtained a good internal consistency reliability (Cronbach's alpha coefficients ranged: 0.76–0.91), good composite reliability (composite reliability ranged: 0.70–0.95) and satisfactory fit of the model (GFI = 0.95; SRMR = 0.05; RMSEA = 0.07). We further performed stratified analysis according to cities, and the index remained stable. Workers with higher scores of WSI-SC2 were more likely to uptake COVID-19 test. This multi-city large study developed a novel and validated tool that could horizontally measure the workplace safety towards SARS-Cov-2 in non-healthcare workers.

## Introduction

Differing from previous severe acute respiratory syndrome (SARS), coronavirus disease 2019 (COVID-19) pandemic caused by severe acute respiratory syndrome coronavirus 2 (SARS-CoV-2) is experiencing with a larger scale of epidemic and faster speed of transmission^1,2^. Although infection control is always highlighted on the occupational safety and health (OSH) agenda in healthcare setting, safe work away from virus also plays an equally important role in non-healthcare settings to maintain healthy and productive workforce, but this was not underlined previously. The World Health Organization (WHO) and the Occupational Safety and Health Administration (OSHA) have recommendations emphasizing on engineering and administrative control measures in keeping safe work practice^3–5^, with some general guidance on infection control towards SARS-Cov-2 such as work from home, social distancing and personal hygiene^3–5^. However, only limited evidence is obtained from scientific research to inform measures of infection control and prevention for COVID-19 in non-healthcare settings. Little has been known about the specific actions needed by the employers and employees from non-healthcare industries to protect themselves from risk of infection. There is an urgent need to strengthen the current guidance to maintain safe workplace against infectious diseases. Until now, there has been lacking of evidence on a validated tool to assess workplace safety towards SARS-Cov-2 for combating COVID-19 in non-healthcare work settings.

Safe and healthy workplace is always advocated to promote production and sustain workforce. Workplace safety towards SARS-CoV-2 tends to become a long-term issue with the viral evolution. Great negative impacts on the prospects of economic development may occur if significant concerns of workplace safety persists, and this is especially important for China who holds the world’s largest labor market derived from a large diversity of industries and professions. We thus raised an important research question about “What are the most significant concerns that are used collectively as a tool to monitor workplace safety towards SARS-Cov-2 in the non-healthcare work settings?” Such initiatives of identifying significant concerns on workplace safety and sustainability triggers a novel research aiming at developing a validated index tool that could reliably measure workplace safety towards SARS-Cov-2 based on a large study comprising of 6684 workers recruited from a variety of non-healthcare industries from three cities of China (Wuhan, Nanjing and Hong Kong) with different social-culture background and economic development. The questionnaire was developed with key domains proposed by occupational health and safety experts and literature review, while supplemented by in-depth interviews with 250 workers from both the management and frontline worker levels. By performing both the exploratory factor analysis and confirmatory factor analysis, this study developed a novel index tool that could be used for comparing and monitoring the changes of workplace safety measures towards SARS-Cov-2 and the related protective behavior over time or between different groups of workers. A warning sign could be provided if the tool measures an outstandingly low score of index in a specific year or a specific work setting to inform policy making of infection control.

## Results

### Descriptive characteristics of the study population

Of the 6684 workers with complete data, over 60% (n = 4274) of participants were male and 58% (n = 3831) were married. Around a quarter of them were aged below 30 (n = 1738, 26.2%), 30–39 (n = 1764, 26.6%) and 40–49 (n = 1848, 27.8%) respectively, but only 2% of them (n = 133) were aged 60 or above. Most of them (n = 6046, 90.5%) were the full-time non-healthcare workers. The most common industry type was “Manufacturing” (n = 1154, 17.3%), followed by “Professional, scientific and technical activities” (n = 925, 13.8%) and “Education” (n = 612, 8.4%). Around 40% of the participants (n = 2648) had even taken COVID-19 testing (Table 1).

**Table 1:** Socio-demographic characteristics of 6684 non-healthcare workers from Hong Kong, Nanjing and Wuhan of China.

Demographic characteristic	n	%
**Sex**		
Male	4274	64.6
Female	2344	35.4
**Age**		
< 30	1738	26.2
30–39	1764	26.6
40–49	1848	27.8
50–59	1159	17.5
≥ 60	133	2.0
**Marital status**		
Married	3831	58.1
Single	2423	36.7
Cohabiting	124	1.9
Divorced/widowed	220	3.3
**Education attainment**		
Middle school or below	1089	16.3
High school	1541	23.1
College diploma	2942	44.0
Undergraduates or above	1027	15.4
Refuse	85	1.3
**Employment status**		
Full-time workers	6046	90.5
Part-time workers	296	4.4
Self-employed	197	3.0
Employer	55	0.8
Refuse	90	1.4
**Industry type**		
Agriculture, forestry and fishing	140	2.1
Manufacturing	1154	17.3
Electricity and gas supply	133	2.0
Construction	227	3.4
Import/export, wholesale and retail trades	381	5.7
Accommodation and food service activities	396	5.9
Transportation, storage, postal and courier services	512	7.7
Information and communications	281	4.2
Financial and insurance activities	132	2.0
Real estate activities	213	3.2
Professional, scientific and technical activities	925	13.8
Administrative and support service activities	287	4.3
Public administration	559	8.4
Education	612	9.2
Human health and social work activities	164	2.5
Arts, entertainment and recreation	148	2.2
Other service activities^1^	420	6.3
**Years of working experiences**		
≤ 5	1574	24.0
5–10	1113	17.0
10–20	1527	23.3
20–30	1334	20.3
> 30	1015	15.5
**History of uptake COVID-19 test**		
No	4036	60.4
Yes	2648	39.6

^1^Other service activities including: Other service activities, Mining and quarrying, Water supply; sewerage, waste management and remediation activities, and Work activities within domestic households industries.

### Exploratory factor analysis

We performed exploratory factor analysis based on the initial random sub-sample (n = 3342) to identify contributing manifest variables to the workplace safety index towards SARS-Cov-2 (WSI-SC2). Varimax rotation was used to identify the domain factors and interpret the correlations between manifest variables. According to the scree plot, three major domain factors seemed to be suitable in this study. We determined all manifest variables if they had factor loadings larger than 0.50 and removed those with factor loadings below a level of 0.50. If the KMO value was 0.91 (mediocre: 0.5–0.7; good: 0.7–0.8; great: 0.8–0.9; excellent > 0.9) and the Bartlett’s test of sphericity’s p-value was < 0.001, it indicated that there are sufficient correlations between the manifest variables. As shown in Supplementary Table 1, the EFA model identified a total of 22 manifest variables with three domain factors to form a novel WSI-SC2, which was comprised of 3 sub-domain factors “Workplace infection control measures and prevention”, “Worker’s personal preventive behavior and awareness towards infectious diseases” and “Company’s occupational safety and health management and commitment”. According to the EFA modeling results, items related to the general guidance and awareness on infection control towards SARS-Cov-2 were not identified as the contributing factors of the novel WSI-SC2.

### Confirmatory factor analysis

We further carried out CFA to confirm the initial results obtained from the EFA model based on another subset of randomly split sample of the 3342 workers. As shown in Table 2, eight manifest variables related to general management and personal general practice that were non-specific to workplace infection control were removed from the model due to the low path coefficient and low composite reliability of the corresponding factors, resulting in 14 manifest variables retained in the CFA modeling. We compared differences in sociodemographic characteristics between workers included in EFA and CFA models, and there was no statistical significance (Supplementary Table 2).

**Table 2:** Descriptive statistics of items in the final model.

Factor	Item	Mean	SD
Workplace infection control measures and prevention	Q2 I think the company's disease prevention measures are effective	3.88	0.95
	Q4 ‘Occupational Safety and Health Ordinance’ ensures my safety and health while working in office	3.77	1.06
	Q11 My employer has provided guidelines and information on disease response in a timely and accurate manner	3.95	0.96
	Q12 My employer/ management has established a disease system to monitor the health of the employees	3.74	1.07
	Q17 The public items in the company are disinfected regularly	3.80	1.08
	Q18 The public facilities, such as air-conditioners, are disinfected regularly	3.75	1.08
	Q20 My company provides enough surgical masks approved with protection standard for the employees	3.82	1.18
	Q21 My company has installed physical barriers between employees or between employees and customers to reduce the risk of disease transmission	3.33	1.23
Company’s occupational safety and health management and commitment	Q7 Employees and management work together to ensure a safe working environment	3.92	1.14
	Q8 The management adopts appropriate control measures when the employees expose to health or safety risks	3.82	1.13
	Q9 The health and safety of the employees is the top priority of management	3.82	1.22
Worker’s personal preventive behavior and awareness towards infectious diseases	Q23 I often clean my hands with soap/ alcohol-based handrub at work	4.32	0.76
	Q28 If my family members or I have been in contact with someone who has COVID-19, I will inform my supervisor immediately	4.36	0.72
	Q29 I try to avoid close contact with colleagues or clients	4.30	0.76

*SD* standard deviation.

We calculated fit indices for measuring the goodness of fits statistics of WSI-SC2 verified by CFA. As shown in Table 3, the model obtained a good model fit based on the GFI (0.059), SRMR (0.04), AGFI (0.91), RMSEA (0.08), Bentler comparative fit index (0.94) and Bentler–Bonett NFI (0.94). We further stratified the model according to cities and almost all models achieved a good-fitting model (Supplementary Table 3).

**Table 3:** Summary of fit indices from confirmatory factor analysis.

	Fit indices	Recommended value	Observed value	Result
Absolute Index	Goodness of Fit Index (GFI)	> 0.9	0.9379	Satisfactory
	Standardized RMR (SRMR)	< 0.08	0.0389	Satisfactory
Parsimony Index	Adjusted GFI (AGFI)	> 0.9	0.9119	Satisfactory
	RMSEA Estimate	< 0.08	0.0765	Satisfactory
Incremental Index	Bentler Comparative Fit Index	> 0.9	0.9383	Satisfactory
	Bentler–Bonett NFI	> 0.9	0.9354	Satisfactory

Regarding the reliability and validity of three domain factors of the newly developed index (i.e., WSI-SC2), Cronbach's α coefficients assessing the internal consistency reliability for “Workplace infection control measures and prevention”, “Company occupational safety and health management and commitment” and “Worker’s personal preventive behavior and awareness towards infectious diseases” are 0.89, 0.91, 0.76, respectively. All of them were above the suggested benchmark of 0.7 and presented a good internal consistency within the manifest variables. Factor loadings of the models ranged from 0.60 to 0.80 for “Workplace infection control measures and prevention”, from 0.83 to 0.92 for “Company’s occupational safety and health management and commitment”, from 0.62 to 0.79 for “Worker’s personal preventive behavior and awareness towards infectious diseases” (Table 4). All values of composite reliability (C.R.) were higher than 0.7 and the Average Variance Extracted (AVE) were higher than 0.5, indicating the reliability of the newly developed index was satisfied.

**Table 4:** Construct reliability test results.

Constructs and indicators	Std. loading	t-value	Pr. > |t|	Reliability	Cronbach's α	C.R.	AVE
**Workplace infection control measures and prevention**					0.886	0.889	0.503
Q2	0.674	64.19	< 0.001	0.455			
Q4	0.658	60.65	< 0.001	0.433			
Q11	0.754	87.38	< 0.001	0.568			
Q12	0.800	107.1	< 0.001	0.640			
Q17	0.741	82.96	< 0.001	0.549			
Q18	0.776	96.13	< 0.001	0.602			
Q20	0.644	57.63	< 0.001	0.415			
Q21	0.601	49.77	< 0.001	0.361			
**Company’s occupational safety and health management and commitment**					0.909	0.950	0.878
Q7	0.885	166.1	< 0.001	0.885			
Q8	0.922	196.3	< 0.001	0.922			
Q9	0.828	126.9	< 0.001	0.828			
**Worker’s personal preventive behavior and awareness towards infectious diseases**					0.763	0.770	0.530
Q23	0.615	45.66	< 0.001	0.379			
Q28	0.772	65.87	< 0.001	0.596			
Q29	0.785	67.55	< 0.001	0.616			

*std* standard, *AVE* average variance extracted, *C.R.* composite reliability.

### WSI-SC2 and selected characteristics

The mean scores of WSI-SC2 and the sub-indices of “Workplace infection control measures and prevention”, “Company’s occupational safety and health management and commitment” and “Worker’s personal exposure prevention behavior and awareness towards infectious diseases” in our sample were 54.7 (SD = 8.8), 30.1 (SD = 6.3), 11.7 (SD = 3.2) and 13.0 (SD = 1.8), respectively. Male participants scored higher on “Workplace infection control measures and prevention” sub-index, but lower on “Company occupational safety and health management and commitment” and “Worker’s personal preventive behavior and awareness towards infectious diseases” sub-index. Participants with higher education tended to have lower “Workplace infection control measures and prevention” sub-index score, but higher “Company’s occupational safety and health management and commitment” and “Worker’s personal preventive behavior and awareness towards infectious diseases” sub-indices scores. In addition, participants who were full-time workers or employers scored higher on WSI-SC2 and the two sub-indices, i.e., “Workplace safety and infection control measures and prevention”, and Worker’s personal exposure preventive on behavior and awareness towards infectious diseases”. We also observed that participants who worked in the following industries scored high in WSI-SC2, they were “agriculture, forestry, and fishing”, “import/export, wholesale and retail trade”, “accommodation and food service activities”, “art, entertainment and recreation”, and “other services activities”. Participants who were younger and had a history of COVID-19 testing were scored higher on WSI-SC2 and all the sub-indices (Supplementary Table 4).

## Discussion

This large multi-city study developed a new index tool to measure the workplace safety towards infection control of SARS-Cov-2 for combating COVID-19 in the non-healthcare workers from Wuhan, Nanjing and Hong Kong of China. All manifest variables of the new WSI-SC2 were initially obtained via a systematic review of the published literature and the guidance and recommendations on workplace prevention of COVID-19 from the World Health Organization^5^, the United States Centres for Disease Control^3,4^ and OSHA^6^. Results from the combined EFA and CFA modelling confirmed that the newly developed WSI-SC2 is a valid and reliable tool to measure the level of workplace safety towards SARS-Cov-2 for combating COVID-19 among non-health workers from diverse industries with varied socioeconomic status.

Among manifest variables finally included in the WSI-SC2, 11 manifest variables were classified to the sub-indices at the company level (i.e., “Workplace infection control measures and prevention”, “Company’s occupational safety and health management and commitment”) and only 3 manifest variables were classified to the sub-index “Worker’s personal exposure prevention behavior and awareness towards infectious diseases”. As more contributing manifest variables came from the sub-domains related to the company level than those from the individual worker’s personal level, it indicates a more important role of the company in promoting workplace safety and infection control than that of the individual worker level. Moreover, this study emphasizes that company authorities should take the workplace as a priority setting as one of the wellness programmes for fighting against infectious diseases by implementing control measures specific to the workplace, while supported by promoting preventive exposure behaviour of the individual workers. The established general guidance and awareness on infection control towards SARS-Cov-2 (e.g., daily temperature screening, flexible sick leave policy, social distancing from coughing or sneezing) was not identified as the contributing manifest variables of the new WSI-SC2 due to their non-specific features to the workplace. To test whether our results are sensitive to culture difference and socioeconomic background, we performed subgroup analysis for Hong Kong, Nanjing and Wuhan, and found all these models achieved good-fitting, suggesting that this newly developed WSI-SC2 is likely to be applied to other cities or countries as a validated tool for comparing workplace safety towards SARS-Cov-2 in non-healthcare work settings.

Female participants in our study and those with higher education attainment had lower score for the sub-index “Workplace infection control measures and prevention”, but higher score for other 2 sub-indices “Company’s occupational safety and health management and commitment” and “Worker’s personal prevention behavior and awareness towards infectious diseases”. In line with the previous studies, more women had higher awareness to adopt precautionary measurements of COVID-19, whereas men were less conscious of health^7–9^. It has been reported that participants with higher education level were more likely to get a better understanding and higher awareness of COVID-19 via self-education^7,8^, which led them more actively to modifying their hazardous exposure behavior but more prone to comply with company’s management of infection control with more commitments. However, they may also be aware of more defects and insufficient workplace infection control measures and leading to the lower score on “Workplace infection control measures and prevention” sub-index. Intriguingly, participants who had ever taken COVID-19 testing were associated with higher scores of WSI-SC2 and the sub-indices. Workers who scored high in “Workplace infection control measures and prevention” sub-index may work in a company with relatively good infection control measures which led them more likely to be encouraged to uptake the COVID-19 testing. Workers with high “Worker’s personal prevention behavior and awareness towards infectious diseases” sub-index may have a high awareness toward COVID-19 and thus they were more willing to uptake COVID-19 testing. A higher WSI-SC2 score in participants employed in “agriculture, forestry, and fishing”, “import/export, wholesale and retail trade”, “accommodation and food service activities”, “art, entertainment and recreation”, and “other services activities” may be related to their higher chances of providing close contact services to the public, and the workers from these industries may have a higher risk of getting infected at work. According to our results in the in-depth interview, the risk perception of workers who had daily contact with large size of the unfamiliar crowds, unhygienic behaviors of clients/customers, and use of public transportation to commute to work had a higher perceived risk of infection than other workers such as those who worked at fixed location in office, and no/little contacts with clients/customers. Thus, workers from these industries may have a higher sense of awareness and protective behaviors against SARS-Cov-2. Additionally, the company from these industries may provide more occupational safety and health management as well as infection control measures and prevention as they are aware that their employees may have a higher chance of getting infected.

The merit of this study is that we developed a novel index tool among workers in a variety of non-healthcare work settings in China. We tested the index separately in different cities with varied socioeconomic development and the tool is stable and robust. We obtained a large sample size that was sufficient for a validation process by taking two separate and comparable samples. This study contains a comprehensive list of manifest variables obtained from a careful systematic review by searching Pubmed and Embase, recommendations and guidance on prevention of COVID-19 from WHO/OSHA/ILO, and qualitative in-depth interviews with about 250 clients. We excluded about 12% participants with missing data containing more male with younger age and lower education level (Supplementary Table 5), which may weaken the representativeness of the sample and led the score of WSI-SC2 to be underestimated by 2–5%. It should cautious that this index was specifically designed to assess the workplace control measures and personal exposure preventive behavior in non-healthcare workers, therefore, the results of this study may not be applicable to nurses and medical doctors in healthcare work settings. Ventilation is now known to be one of the protective measures against COVID-19 in the workplace but it was not finally included in our survey. During the pilot test, we included a question about whether the window in workplace was open with natural ventilation, whether the window was closed but with general ventilation, however, we got a high missing rate on this ventilation related question. It may be because the participants cannot distinguish whether their workplace has good ventilation or not such as a shopping mall or a restaurant. Lastly, some AI methods have been used to detect face masks in different places (e.g., crowded places, indoors or public places)^10^. It would be very interesting if the component of using AI methods could be integrated into our newly developed index tool to improve the performance of the workplace safety index towards SARS-Cov-2, however, this scope of research will be addressed in the future study.

## Conclusions

Evidence from the first year survey from 3 cities of China demonstrated that the newly developed index is a validated tool for horizontally measuring the workplace safety towards infection control of SARS-Cov-2 for combating the COVID-19 among non-healthcare workers. Compared with personal preventive exposure behavior, this study suggests that workplace infection control measures and company’s OSH management have played more important roles in combating the COVID-19. This study further emphasizes the importance of company’s leadership and management in implementation of control measures for COVID-19. Nevertheless, evidence on whether the tool is also valid for longitudinally monitoring will soon be available from the repeated measurement in the second year.

## Methods

### Study sample and study site

This report presented findings from the first year survey from a repeated industry-based multicenter cross-sectional study in Wuhan (Hubei Province), Nanjing (Jiangsu Province) and Hong Kong of China during 07/2020–04/2021. We chose these 3 cities to represent different socioeconomic background in China with unique geographic variations. Wuhan is a city located in Hubei Province in Central China accommodating 11 million people, which is the epicentre of COVID-19 in the early spread of SARS-Cov-2^11^. Nanjing, another city located in the east of Wuhan and close to Shanghai, has 329 local confirmed cases among 8.4 million population as of Oct 2021^12^. Hong Kong is a city much smaller than Wuhan and Nanjing but it is the most developed and crowded area worldwide, and as of Oct 2021 there have been 12,347 local cases identified from a 7-million population^13^. While there exist differences in cultural background and economic development, these 3 cities have some common types of industries which led to a reliable comparability of workplace safety and prevention measures towards SARS-Cov-2 between cities.

Strategically, we sought collaborations through the network of the Hong Kong Federation of Trade Unions-Occupational Safety and Health Association and the Occupational Disease Prevention Hospital of Nanjing and Wuhan site to invite workers participating in this study, supported by internet-based survey and companies to increase sample size. A total of 7596 non-healthcare workers aged 18 years old or above were recruited from 3 cities and responded the standardized questionnaires containing information on sociodemographic (e.g., age, sex, educational attainment, marital status), job-related information (employment status, employment years and industry type), and history of COVID-19 testing. Overall, 6684 respondents had complete data and were finally included in this report.

This study was approved by the Survey and Behavioral Research Ethics (Reference No. SBRE-19-792) of the Chinese University of Hong Kong, Ethics and Human Subject Committee of Nanjing Medical University (Reference No.: [2020]554) and Tongji Medical College, Huazhong University of Science and Technology from Wuhan (Reference No.: [2020]S212). The study was performed in accordance with the Declaration of Helsinki. Informed consents from participants have been obtained before they started the questionnaires.

### Questionnaire development

There is no validated measurement to assess the workplace safety issues and worker’s personal preventive behaviour towards SARS-Cov-2 in non-healthcare work settings. In the phase of questionnaire design, we designed relevant questions using similar methodology of developing a novel index tool of safety culture measurement^14^. Specifically, the questionnaire was developed with key domains proposed by occupational health and safety expert working group of Wuhan, Nanjing and Hong Kong after conducting a systematic review through searching published papers using subheading “COVID-19”, “SARS-Cov-2”, “workplace”, “industries”, and “occupation” from PubMed and Embase for the period 11/2019–4/2020^15,16^. Pertinent studies were obtained to retrieve questions related to the proposed key domains covering company management and commitment of OSH and infection control, safety culture and worker’s personal exposure behaviors towards infection control including COVID-19. Moreover, relevant recommendations from World Health Organization^5^, the United States Centres for Disease Control and Prevention^3,4^, and OSHA were also sought and reviewed^6^. The draft questionnaire was enhanced by additional questions obtained from the parallel in-depth qualitative interviews among approximate 250 management and workers from 3 cities through transcribing the audios recoded from each in-depth interview and then categorizing them by themes following the Grounded Theory approach, including knowledge of COVID-19, personal prevention behaviors against COVID-19, risk perception of COVID-19 infection, workplace safety measures, workplace risk management and overall evaluation. The enhanced questionnaire was further reviewed by four external experts specialized in occupational and environmental medicine, occupational hygiene, occupational nurse, infectious diseases and public health. The finalized questionnaire contains 40 manifest variables focusing on 4 potential domains covering workplace infection control measures and prevention, worker’s personal preventive behaviour and awareness towards infectious diseases, company’s occupational safety and health management and commitment, and the general guidance and awareness on infection control towards SARS-Cov-2. Each manifest variable under the corresponding domain was designed with a 5-point Linkert scale, i.e. (1) strongly disagree (2) disagree (3) neither agree nor disagree (4) agree (5) strong agree, and each participant was asked to indicate his/her level of agreement for each manifest variable.

### Statistical analysis

Descriptive statistics were used to describe the demographic characteristics of participants enrolled in this study. We calculated mean scores and standard deviations for each manifest variable. We then randomly split the whole dataset into two halves of equal size as training dataset and validation dataset, using a SAS procedure called “proc surveyselect”. We used equal probability sampling methods to select these datasets, and invoked the procedure separately for each city. Participants with incomplete data were excluded from further analysis. To assess the suitability of performing factor analysis, we conducted Bartlett’s test of sphericity and calculated Kaiser–Meyer–Olkin (KMO) measure of sample adequacy. A small value (i.e. less than 0.05) of the significance level in Bartlett's test of sphericity and high KMO value (i.e. close to 1.0) indicates the good matrix identity and sampling adequacy for factor analysis^17,18^.

Exploratory factor analysis (EFA) is a discriminant analysis to examine the underlying dimensionality of the manifest variables^19^. EFA was conducted on the training dataset using maximum likelihood estimation to identify the latent factors that explain the common variance of the manifest variables. The number of potential factors was determined by the scree plot and the principles of simple structure^20,21^. The factors with relatively large eigenvalues were retained for rotation. An orthogonal varimax rotation was performed to obtain the rotated factor matrix^22^. Manifest variables with factor loadings greater than 0.50 were considered acceptable for being retained in a new collective factor or domain. We then performed confirmatory factor analysis (CFA) on the validation dataset to verify the factor structure derived from the EFA, and also used to confirm the prespecified relationship identified by EFA and further evaluate and refine the index derived from the EFA^18^. Multiple goodness-of-fit indices were used to evaluate the model fit of CFA, including goodness-of-fit index (GFI), standardized root mean square residual (SRMR), adjusted GFI (AGFI), root mean square error of approximation (RMSEA), comparative fit index (CFI), and normed fit index (NFI). Values of GFI/AGFI/CFI/NFI ≥ 0.90 and SRMR/RMSEA ≤ 0.08 were considered as indicating acceptable model fit^23–25^. We further evaluated the model fit by different cities (i.e. Hong Kong, Nanjing, and Wuhan) to elaborate the robustness of factors finally retained in the model.

Internal consistency of the factors retained in the final model was evaluated using Cronbach’s α coefficient and composite reliability (C.R.). Values of Cronbach’s α coefficient and C.R. greater than 0.70 were considered acceptable^26^. To ensure good internal consistency of each factor, we discarded manifest variables with low path coefficients. In addition, we adopted the average variance extracted (AVE) to assess the amount of variance captured by factors, and the value of AVE greater than 0.50 indicates that the variance captured by the factor is larger than measurement error^27^.

We used the manifest variables that were retained in the final model to develop the new index and sub-indices, and the index reflected levels of workplace safety towards COVID-19 among non-healthcare workers. The total scores of the index and sub-indices were calculated by summation of the corresponding manifest variables, however, there is cautious that the new index is suggestive while the COVID-19 pandemic may not equally affect all characteristics particularly for people with different socioeconomic background and health challenges. We performed independent t-test and one-way ANOVA to compare the differences in the index and the sub-indices by the selected characteristics including sex, age, educational attainment and history of uptake COVID-19 testing. All statistical analyses were performed using SAS 9.4 (SAS Institute, Cary, NC).

## Supplementary Information

Supplementary Tables.

## Data Availability

The datasets used and/or analysed during the current study related Workplace safety index towards SARS-Cov-2 to available from the corresponding author on request.

## Abbreviations

SARS: Severe acute respiratory syndrome
COVID-19: Coronavirus disease 2019
SARS-Cov-2: Severe acute respiratory syndrome coronavirus 2
OSH: Occupational safety and health
WHO: World Health Organization
KMO: Kaiser–Meyer–Olkin
EFA: Exploratory factor analysis
CFA: Confirmatory factor analysis
GFI: Goodness-of-fit index
SRMR: Standardized root mean square residual
RMSEA: Root mean square error of approximation
CFI: Comparative fit index
NFI: Normed fit index
C.R.: Composite reliability
AVE: Average variance extracted
WSI-SC2: Workplace safety index towards SARS-Cov-2
