# Supplementary material for: Determinants of workplace safety towards SARS-Cov-2 and combating COVID-19 among non-healthcare workers in Hong Kong, Nanjing and Wuhan, China
Source: Sci Rep. 2022 Sep 9;12:15249. doi: 10.1038/s41598-022-19195-4 (PMC9462604; doi:10.1038/s41598-022-19195-4)
Supplement: Supplementary file 1 — Supplementary Tables. [file 41598_2022_19195_MOESM1_ESM.pdf]

Supplementary table 1. Factor load matrix according to exploratory factor analysis

| Manifest variables | Factor1 | Factor2 | Factor3 |
|---|---|---|---|
| Domain factor 1 - Workplace infection control measures and prevention | | | |
| Q12 My employer/ management has established a disease system to monitor the health of the employees. | **0.76** | 0.03 | 0.1 |
| Q18 The public facilities, such as air-conditioners, are disinfected regularly. | **0.76** | 0.08 | 0.11 |
| Q11 My employer has provided guidelines and information on disease response in a timely and accurate manner. | **0.71** | 0.12 | 0.15 |
| Q17 The public items in the company are disinfected regularly. | **0.69** | 0.13 | 0.11 |
| Q20 My company provides enough surgical masks approved with protection standard for the employees. | **0.66** | 0.09 | 0.11 |
| Q2 I think the company's disease prevention measures are effective. | **0.64** | 0.04 | 0.13 |
| Q4 ‘Occupational Safety and Health Ordinance’ ensures my safety and health while working in office. | **0.62** | -0.01 | 0.09 |
| Q21 My company has installed physical barriers between employees or between employees and customers to reduce the risk of disease transmission. | **0.62** | -0.02 | 0.07 |
| Q16 Our company reduced air-conditioning during the COVID-19 outbreak. | **0.60** | -0.18 | 0.04 |
| Q13 My employer/ management adopts a flexible sick leave policy during the COVID-19 outbreak. | **0.57** | 0.11 | 0.11 |
| Q22 During the COVID-19 pandemic, our company suggests to have virtual meeting. | **0.57** | 0.2 | 0.14 |
| Q19 The company has introduced daily temperature screening to the employees before work. | **0.56** | 0.14 | 0.05 |
| Domain factor 2 - Worker’s personal preventive behavior and awareness towards infectious diseases | | | |
| Q29 I try to avoid close contact with colleagues or clients. | 0.27 | **0.63** | 0.02 |
| Q23 I often clean my hands with soap/ alcohol-based handrub at work. | 0.29 | **0.62** | 0.04 |
| Q28 If my family members or I have been in contact with someone who has COVID-19, I will inform my supervisor immediately. | 0.3 | **0.61** | 0.08 |
| Q55 I usually clean my hands with alcohol-based handrub. | 0.15 | **0.56** | 0.02 |
| Q53 Social distancing is very important to prevent the outbreak of COVID-19 in the community. | 0.13 | **0.51** | 0 |
| Q51 After using toilet, I put the toilet lid down before flushing. | -0.05 | **0.51** | -0.06 |
| Q59 I keep a distance from someone who is coughing or sneezing | 0.18 | **0.50** | 0.03 |
| Domain factor 3 - Company occupational safety and health management and commitment | | | |
| Q7 Employees and management work together to ensure a safe working environment. | 0.16 | 0.11 | **0.88** |
| Q8 The management adopts appropriate control measures when the employees expose to health or safety risks. | 0.22 | 0.12 | **0.88** |
| Q9 The health and safety of the employees is the top priority of management. | 0.17 | 0.14 | **0.80** |

Supplementary table 2 Demographic information of participants between exploratory factor analysis and confirmatory factor analysis

| Demographic characteristics | Participants in EFA | | Participants in CFA | |
|---|---|---|---|---|
| | n | % | n | % |
| Gender | | | | |
| Male | 2143 | 64.6 | 2131 | 64.6 |
| Female | 1177 | 35.5 | 1167 | 35.4 |
| Age | | | | |
| <30 | 873 | 26.3 | 865 | 26.1 |
| 30-39 | 865 | 26.0 | 899 | 27.1 |
| 40-49 | 928 | 27.9 | 920 | 27.7 |
| 50-59 | 594 | 17.9 | 565 | 17.0 |
| ≥60 | 66 | 2.0 | 67 | 2.0 |
| Marital Status | | | | |
| Married | 1931 | 58.5 | 1900 | 57.6 |
| Single | 1180 | 35.8 | 1243 | 37.7 |
| Cohabiting | 77 | 2.3 | 47 | 1.4 |
| Divorced / Widowed | 111 | 3.4 | 109 | 3.3 |
| Education attainment | | | | |
| Middle School or below | 545 | 16.3 | 544 | 16.3 |
| High School | 739 | 22.1 | 802 | 24.0 |
| College Diploma | 1494 | 44.7 | 1448 | 43.3 |
| University or above | 516 | 15.4 | 511 | 15.3 |
| Refuse | 48 | 1.4 | 37 | 1.1 |
| Employment Status | | | | |
| Full time workers | 3017 | 90.3 | 3029 | 90.6 |
| Part-time workers | 149 | 4.5 | 147 | 4.4 |
| Self-employed | 100 | 3.0 | 97 | 2.9 |
| Employer | 25 | 0.8 | 30 | 0.9 |
| Refuse | 51 | 1.5 | 39 | 1.2 |
| Industry types | | | | |
| Agriculture, forestry and fishing | 62 | 1.9 | 78 | 2.3 |
| Manufacturing | 583 | 17.4 | 571 | 17.1 |
| Electricity and gas supply | 57 | 1.7 | 76 | 2.3 |
| Construction | 107 | 3.2 | 120 | 3.6 |
| Import/export, wholesale and retail trades | 192 | 5.7 | 189 | 5.7 |
| Accommodation and food service activities | 196 | 5.9 | 200 | 6.0 |
| Transportation, storage, postal and courier services | 271 | 8.1 | 241 | 7.2 |
| Information and communications | 13 | 4.0 | 146 | 4.3 |
| Financial and insurance activities | 59 | 1.8 | 73 | 2.2 |
| Real estate activities | 110 | 3.3 | 103 | 3.1 |
| Professional, scientific and technical activities | 450 | 13.5 | 475 | 14.2 |

| | | | | |
|---|---|---|---|---|
| Administrative and support service activities | 143 | 4.3 | 144 | 4.3 |
| Public administration | 282 | 8.4 | 277 | 8.3 |
| Education | 311 | 9.3 | 301 | 9.0 |
| Human health and social work activities | 84 | 2.5 | 80 | 2.4 |
| Arts, entertainment and recreation | 72 | 2.2 | 76 | 2.3 |
| Other service activities | 228 | 6.8 | 192 | 5.8 |
| Years of working experience | | | | |
| ≤5 | 809 | 24.21 | 782 | 23.40 |
| 5-10 | 573 | 17.15 | 552 | 16.52 |
| 10-20 | 745 | 22.29 | 790 | 23.64 |
| 20-30 | 672 | 20.11 | 666 | 19.93 |
| >30 | 508 | 15.20 | 509 | 15.23 |
| Refuse | 35 | 1.05 | 43 | 1.29 |
| COVID-19 test | | | | |
| No | 2016 | 60.63 | 1989 | 59.77 |
| Yes | 1709 | 39.37 | 1339 | 40.23 |

Abbreviation: EFA, exploratory factor analysis; CFA, confirmatory factor analysis

[1] Other service activities including: Other service activities, Mining and quarrying, Water supply; sewerage, waste management and remediation activities, and Work activities within domestic households industries.

Supplementary table 3 Summary of fit indices from confirmatory factor analysis by cities

| | Fit Indices | Hong Kong | Nanjing | Wuhan |
|---|---|---|---|---|
| Absolute Index | Goodness of Fit Index (GFI) | 0.9521 | 0.8846 | 0.8918 |
| | Standardized RMR (SRMR) | 0.0472 | 0.0555 | 0.0518 |
| Parsimony Index | Adjusted GFI (AGFI) | 0.9321 | 0.8363 | 0.8464 |
| | RMSEA Estimate | 0.0652 | 0.1067 | 0.1011 |
| Incremental Index | Bentler Comparative Fit Index | 0.9192 | 0.8967 | 0.9129 |
| | Bentler-Bonett NFI | 0.9123 | 0.8931 | 0.9092 |

Supplementary table 4 Distribution of "Workplace safety towards SARS-Cov-2 & COVID-19 index" by selected characteristics

| Characteristics | "Workplace safety towards SARS-Cov-2 & COVID-19" index | "Workplace safety and infection control measures and prevention" sub-index | "Company's occupational safety and health management and commitment" sub-index | "Worker's personal exposure preventive on behavior and awareness towards infectious diseases" sub-index |
|---|---|---|---|---|
| | **Mean (SD)** | **Mean (SD)** | **Mean (SD)** | **Mean (SD)** |
| Sex | | | | |
| Male | 55.1 (8.8) | 30.7 (6.1) | 11.6 (3.4) | 12.8 (1.9) |
| Female | 53.9 (8.7) | 28.8 (6.5) | 11.8 (2.9) | 13.3 (1.6) |
| p-value | <0.001 | <0.001 | 0.002 | <0.001 |
| Age | | | | |
| <30 | 55.3 (9.5) | 30.5 (6.7) | 11.8 (3.2) | 13.0 (1.8) |
| 30-39 | 54.0 (9.5) | 29.4 (6.8) | 11.6 (3.2) | 13.0 (1.8) |
| 40-49 | 54.9 (8.0) | 30.3 (5.9) | 11.6 (3.2) | 13.0 (1.8) |
| 50-59 | 54.3 (7.9) | 30.0 (5.9) | 11.5 (3.1) | 12.8 (1.9) |
| ≥60 | 53.4 (6.9) | 28.7 (5.5) | 12.1 (2.8) | 12.6 (1.8) |
| p-value | <0.001 | <0.001 | 0.023 | 0.001 |
| Marital Status | | | | |
| Married | 55.4 (8.4) | 30.8 (5.9) | 11.6 (3.3) | 13.0 (1.8) |
| Single | 53.8 (9.3) | 29.0 (6.8) | 11.7 (2.9) | 13.0 (1.8) |
| Cohabiting | 53.6 (9.3) | 29.1 (6.3) | 11.5 (3.3) | 13.1 (1.9) |
| Divorced / Widowed | 53.6 (9.0) | 29.3 (6.4) | 11.4 (3.4) | 12.9 (2.0) |
| p-value | <0.001 | <0.001 | 0.245 | 0.524 |
| Education attainment | | | | |
| Middle School or below | 56.6 (8.7) | 32.2 (5.4) | 11.7 (3.5) | 12.8 (2.1) |
| High School | 55.3 (9.1) | 30.7 (6.3) | 11.8 (3.2) | 12.8 (1.9) |
| College Diploma | 53.9 (8.9) | 29.3 (6.6) | 11.4 (3.2) | 13.1 (1.7) |
| University or above | 53.8 (7.7) | 28.7 (5.6) | 12.0 (2.5) | 13.0 (1.6) |
| p-value | <0.001 | <0.001 | <0.001 | <0.001 |
| Employment status | | | | |
| Full-time workers | 54.8 (8.8) | 30.2 (6.4) | 11.6 (3.2) | 13.0 (1.8) |
| Part-time workers | 52.8 (9.2) | 28.4 (6.3) | 11.7 (2.8) | 12.7 (1.9) |
| Self-employed | 52.9 (8.4) | 28.5 (6.2) | 11.6 (2.7) | 12.8 (1.8) |

| | | | | |
|---|---|---|---|---|
| Employer | 54.7 (7.6) | 29.5 (5.1) | 12.2 (2.8) | 13.1 (1.9) |
| p-value | <0.001 | <0.001 | 0.639 | 0.007 |
| Industry type | | | | |
| Agriculture, forestry and fishing | 57.0 (9.0) | 32.2 (5.4) | 12.7 (2.4) | 12.1 (2.5) |
| Manufacturing | 56.2 (8.0) | 32.2 (5.2) | 11.2 (3.7) | 12.8 (1.8) |
| Electricity and gas supply | 57.5 (8.5) | 32.4 (5.4) | 12.5 (2.7) | 12.5 (1.9) |
| Construction | 53.1 (8.3) | 29.9 (6.6) | 10.3 (4.1) | 13.0 (1.9) |
| Import/export, wholesale and retail trades | 54.0 (9.3) | 29.1 (6.9) | 11.9 (3.0) | 13.0 (1.9) |
| Accommodation and food service activities | 58.3 (9.7) | 32.8 (6.0) | 12.3 (3.4) | 13.2 (2.0) |
| Transportation, storage, postal and courier services | 54.4 (9.1) | 30.6 (5.8) | 11.2 (3.6) | 12.6 (2.0) |
| Information and communications | 50.9 (8.1) | 26.7 (6.2) | 11.2 (2.9) | 13.0 (1.7) |
| Financial and insurance activities | 50.9 (7.5) | 26.4 (5.5) | 11.3 (2.7) | 13.2 (1.5) |
| Real estate activities | 54.4 (8.8) | 30.3 (6.6) | 11.1 (3.5) | 13.0 (1.8) |
| Professional, scientific and technical activities | 53.4 (8.8) | 28.5 (6.7) | 11.8 (2.8) | 13.1 (1.7) |
| Administrative and support service activities | 55.0 (8.6) | 30.5 (6.4) | 11.4 (3.2) | 13.1 (1.7) |
| Public administration | 52.2 (8.7) | 27.7 (6.5) | 11.4 (2.9) | 13.1 (1.7) |
| Education | 54.7 (7.6) | 29.5 (5.5) | 12.2 (2.4) | 13.0 (1.6) |
| Human health and social work activities | 53.9 (7.3) | 28.7 (5.5) | 11.8 (2.5) | 13.4 (1.6) |
| Arts, entertainment and recreation | 54.5 (9.4) | 29.1 (6.9) | 12.3 (2.3) | 13.1 (1.8) |
| Other service activities | 57.5 (9.5) | 31.7 (6.3) | 12.7 (2.7) | 13.1 (2.0) |
| p-value | <0.001 | <0.001 | <0.001 | <0.001 |
| History of uptake COVID-19 test | | | | |
| No | 52.8 (8.4) | 28.6 (6.5) | 11.2 (3.2) | 13.0 (1.7) |
| Yes | 57.6 (8.6) | 32.3 (5.3) | 12.4 (3.1) | 12.9 (1.9) |
| p-value | <0.001 | <0.001 | <0.001 | 0.026 |

Abbreviation: EFA, exploratory factor analysis; CFA, confirmatory factor analysis

[1] Other service activities including: Other service activities, Mining and quarrying, Water supply; sewerage, waste management and remediation activities, and Work activities within domestic households industries.

Supplementary table 5 Demographic characteristics of participants with and without complete data

| Demographic characteristics | Participants with complete data | | Participants with incomplete data | |
|---|---|---|---|---|
| | n | % | n | % |
| Gender | | | | |
| Male | 4274 | 64.6 | 683 | 81.0 |
| Female | 2344 | 35.4 | 160 | 19.0 |
| Age | | | | |
| <30 | 1738 | 26.2 | 303 | 36.68 |
| 30-39 | 1764 | 26.6 | 247 | 29.90 |
| 40-49 | 1848 | 27.8 | 163 | 19.73 |
| 50-59 | 1159 | 17.5 | 85 | 10.29 |
| ≥60 | 133 | 2.0 | 28 | 3.39 |
| Marital Status | | | | |
| Married | 3831 | 58.1 | 515 | 59.06 |
| Single | 2423 | 36.7 | 322 | 36.93 |
| Cohabiting | 124 | 1.9 | 13 | 1.49 |
| Divorced / Widowed | 220 | 3.3 | 22 | 2.52 |
| Education Level | | | | |
| Middle School or below | 1089 | 16.3 | 334 | 36.62 |
| High School | 1541 | 23.1 | 323 | 35.42 |
| College Diploma | 2942 | 44.0 | 151 | 16.56 |
| Undergraduates or above | 1027 | 15.4 | 41 | 4.50 |
| Refuse | 85 | 1.3 | 63 | 6.91 |
| Employment Status | | | | |
| Full-time workers | 6046 | 90.5 | 759 | 83.22 |
| Part-time workers | 296 | 4.4 | 86 | 9.43 |
| Self-employed | 197 | 3.0 | 29 | 3.18 |
| Employer | 55 | 0.8 | 0 | 0.00 |
| Refuse | 90 | 1.4 | 38 | 4.17 |
| Industry type | | | | |
| Agriculture, forestry and fishing | 140 | 2.1 | 89 | 9.8 |
| Mining and quarrying | 6 | 0.1 | 0 | 0.0 |
| Manufacturing | 1154 | 17.3 | 284 | 31.1 |
| Electricity and gas supply | 133 | 2.0 | 49 | 5.4 |
| Water supply; sewerage, waste management and remediation activities | 36 | 0.5 | 1 | 0.1 |
| Construction | 227 | 3.4 | 37 | 4.1 |
| Import/export, wholesale and retail trades | 381 | 5.7 | 45 | 4.9 |
| Accommodation and food service activities | 396 | 5.9 | 11 | 1.2 |
| Transportation, storage, postal and courier services | 512 | 7.7 | 65 | 7.1 |
| Information and communications | 281 | 4.2 | 8 | 0.9 |
| Financial and insurance activities | 132 | 2.0 | 2 | 0.2 |
| Real estate activities | 213 | 3.2 | 10 | 1.1 |

| | | | | |
|---|---|---|---|---|
| Professional, scientific and technical activities | 925 | 13.8 | 87 | 9.5 |
| Administrative and support service activities | 287 | 4.3 | 3 | 0.3 |
| Public administration | 559 | 8.4 | 21 | 2.3 |
| Education | 612 | 9.2 | 40 | 4.4 |
| Human health and social work activities | 164 | 2.5 | 7 | 0.8 |
| Arts, entertainment and recreation | 148 | 2.2 | 18 | 2.0 |
| Other service activities | 420 | 6.2 | 136 | 14.9 |
| Work activities within domestic households | 2 | 0.0 | 0 | 0.0 |
| Others | 29 | 0.4 | 9 | 1.0 |
| Years of working experience | | | | |
| ≤5 | 1591 | 23.80 | 257 | 28.18 |
| 5-10 | 1125 | 16.83 | 199 | 21.82 |
| 10-20 | 1535 | 22.97 | 216 | 23.68 |
| 20-30 | 1338 | 20.02 | 137 | 15.02 |
| >30 | 1017 | 15.22 | 64 | 7.02 |
| Refuse | 78 | 1.17 | 39 | 4.28 |
| COVID-19 test | | | | |
| No | 4005 | 60.20 | 161 | 18.38 |
| Yes | 2648 | 39.80 | 715 | 81.62 |
